# An evaluation of the EPOS selective chemistry analyser

**DOI:** 10.1155/S1463924687000142

**Published:** 1987

**Authors:** G. J. Hughes, D. J. Wright

**Affiliations:** Department of Biochemistry Coventry and Warwickshire Hospital Stoney Stanton Road Coventry CV1 4FH UK


**Journal of Automatic Chemistry**/*Journal of Clinical Laboratory Automation*, **Vol. 9, No. 2 (April–June 1987), pp. 59–65**

### An evaluation of the EPOS selective chemistry analyser

**G. J. Hughes and D. J. Wright**
*Department of Biochemistry, Coventry and Warwickshire Hospital, Stoney Stanton Road, Coventry CV1 4FH, UK*

### Introduction

The Eppendorf Patient Oriented System (EPOS) is an automated discrete analyser which can perform tests on batches of up to 200 specimens at a rate of up to 300 specimens/h. This high throughput is achieved because of a novel time-sharing system which allows the simultaneous dispensing, incubation and measurement of samples in different cuvettes. The analyser can perform end-point, kinetic, turbidimetric and enzyme immunometric assays which allows a wide range of analytes to be measured. Other features of the EPOS include a comprehensive manual function capability, a low daily maintenance requirement and a self-contained water supply and waste drainage system so that external plumbing is not required.

Figure 1 shows a diagram of the rotor area of the instrument and the major features of the sample and reagent-dispensing systems. Samples for analysis are contained in 2 ml disposable cups with snap-on caps which are placed in numbered positions (links) in a flexible carrier chain. This chain is split into detachable segments, each containing 10 links, which can be joined in any order because the EPOS can read the position of any programmed sample. During the analysis the appropriate samples are moved to the sampling position and the required amount of sample is aspirated by the sample dispenser which pierces the cap of the sample container.

Reagents are contained in one or both of two detatchable reservoirs. The first reagent (R1) reservoir, which is used alone in one reagent chemistries, can hold up to 80 ml of reagent and is situated adjacent to the sample and R1 dispensers. It is covered by a lid which contains an access hole for the R1 dispenser, along with nine wells which are used to hold standards and controls and are accessible by the sample dispenser. The lid also has a small well accessible by the R1 dispenser which contains a detergent solution used to clean the cuvettes at the end of each batch, i.e. when all the designated samples for a particular analysis have been measured. The second reagent (R2) reservoir has a capacity of 20 ml and is used

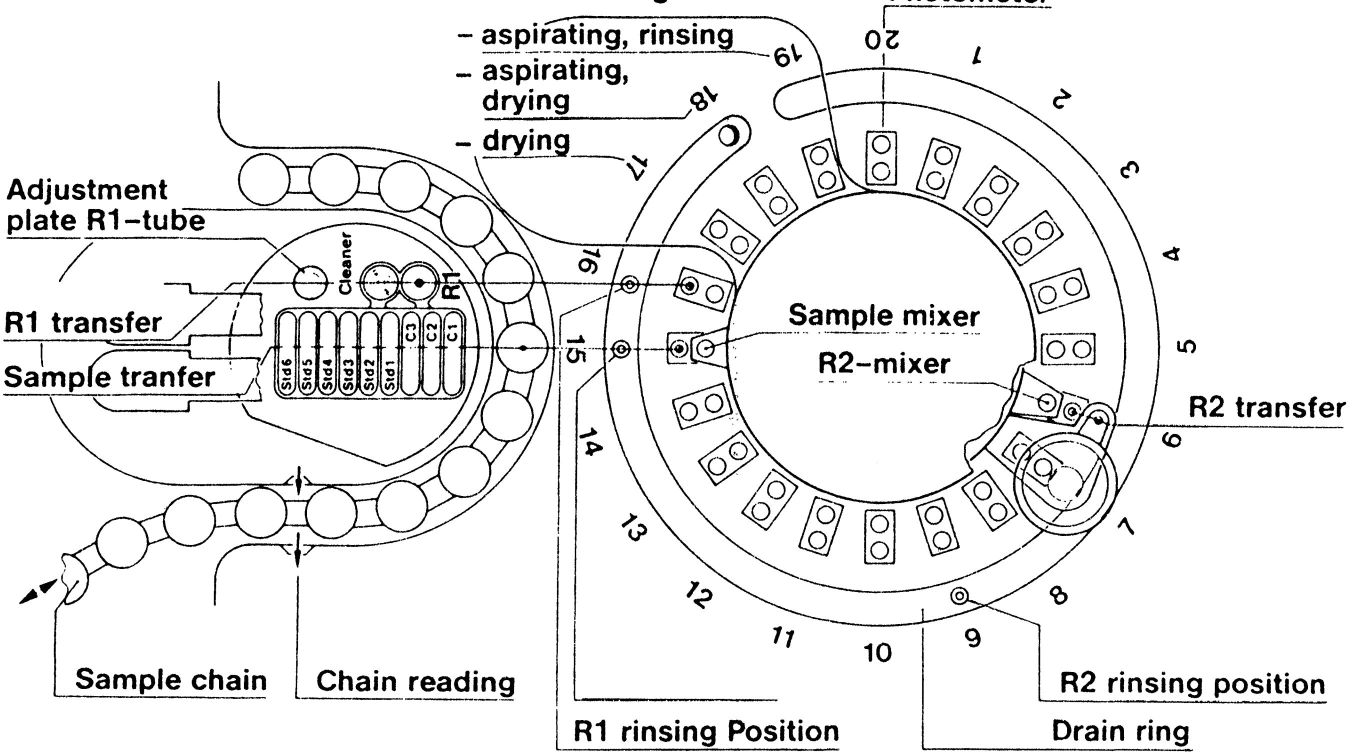

*Figure 1. Features of the dispensing/cuvette rotor area of the EPOS.*

with chemistries which are initiated by reagent addition. The R2 reservoir and dispenser can be situated in any of 10 positions around the rotor circumference which allows greater flexibility for the timing of second reagent addition.

The reactions for all the chemistries take place in 20 'U' shaped cuvettes situated in a heated rotor which can be set to any temperature between 20 and 40 °C in 1 °C intervals. Mixing of sample and reagents is effected by pulsed air pressure at radiowave frequencies directed into one end of the open-ended cuvettes. Mixers are situated adjacent to the sample and second reagent dispensing positions and operate one rotor cycle after the addition of sample and second reagent respectively.

The control of preincubation, incubation, end-point and kinetic measuring times is handled by an integral microprocessor. This optimizes the time of reagent addition, rotor mobility, and measurement interval so that the fastest possible throughput of tests is achieved.

The measuring system in the EPOS consists of a photometer unit which measures the absorbances across the bottom of the 'U' shaped cuvettes. The light source is a mercury vapour lamp associated with double interference filters which select wavelengths of 334 nm, 405 nm, 492 nm, 492 nm, 546 nm, and 578 nm. The photometer will measure a single end-point absorbance, an absorbance change in a single cuvette over a fixed period, or absorbance changes in a number of cuvettes by integrating the accumulated absorbance readings in individual cuvettes every time they pass the measuring position. In many chemistries reagent and sample blanks can be automatically subtracted from the absorbance readings and there is a facility to adjust for high background absorbance where necessary. The EPOS calculates the results from the absorbance values using calibration factors derived from measured standards. It can perform calibrations using a single standard or up to as many as six in the form of a standard line or curve. An integral printer displays the absorbances of the standards along with the results of the tests.

After passing the measuring position the cuvettes pass through a washing station where they are automatically washed, rinsed and dried so that they are available for use again during a large batch. At the end of a batch all the cuvettes are rinsed and dried before being left filled with a dilute detergent solution.

Control of all functions on the instrument is through an integral keyboard. Using this, method parameters can be set, stored methods called up and initiated and a wide range of manual functions controlled. A 16-character LED display next to the keyboard provides a continual record of the instrument status, as well as warning of system errors as they occur.

The aim of this study was to perform a mechanical and clinical evaluation of the EPOS using the guidelines of Broughton *et al.* [1]. The clinical evaluation was carried out on three selected chemistries: calcium, urea and gamma-glutamyl transpeptidase (GGT).

### Precision and accuracy of dispensers

The precision and accuracy of the three dispensers at different volume settings was tested by measuring the absorbances at 334 nm of 20 replicate dilutions of a 1·0 g/l solution of potassium dichromate. For the sample and R2 dispensers, volumes of the potassium dichromate solution sampled by these were mixed in the cuvettes with a fixed volume of 0·005 M sulphuric acid from the R1 dispenser. For the R1 dispenser, volumes of sulphuric acid were used to dilute fixed volumes of potassium dichromate from the sample probe. The mean absorbance of 20 replicate dilutions multiplied by the appropriate dilution factor was then compared with the measured absorbance of a 1·0 g/l potassium dichromate solution and the difference taken to be that between the nominal and actual volumes dispensed.

The results show good precision and accuracy for all three dispensers (see table 1).

*Table 1. Precision and accuracy of dispensing system.*
*(a) Sample dispenser.*

| Volume set (μl) | Accuracy Measured vol. (μl) | Precision CV(%) |
|---|---|---|
| 2·5 | 2·6 | 0·7 |
| 5·0 | 5·2 | 0·4 |
| 10·0 | 10·2 | 0·2 |
| 20·0 | 21·1 | 0·2 |
| 50·0 | 50·5 | 0·3 |

*(b) R1 dispenser.*

| Volume set (μl) | Accuracy Measured vol. (μl) | Precision CV (%) |
|---|---|---|
| 200 | 202 | 0·14 |
| 225 | 228 | 0·20 |
| 250 | 255 | 0·30 |
| 275 | 271 | 0·30 |
| 300 | 307 | 0·30 |

*(c) R2 dispenser.*

| Volume set (μl) | Accuracy Measured vol. (μl) | Precision CV (%) |
|---|---|---|
| 10 | 9·8 | 0·5 |
| 20 | 19·5 | 0·4 |
| 30 | 29·3 | 0·3 |
| 50 | 48·4 | 0·2 |

### Temperature control

The cuvette temperature is controlled by the heating and cooling of the rotor in which they are situated. An integral thermistor measures the temperature of the rotor and its read-out can be displayed. A thermistor that could be inserted into the cuvettes was used to measure the temperature of the liquid inside them. This showed that there was an excellent correlation between the indicated and measured cuvette temperatures ($y = 0{\cdot}982x + 0{\cdot}52$, $r = 0{\cdot}9997$) with negligible between-day (coefficient of variation [CV] = 0·15%) and cuvette-cuvette (CV = 0·1%) temperature variations between 25 and 37 °C. In addition, the time taken for the cuvettes to attain a new temperature between method changes was found to be within the limits quoted by the manufacturer (6 min between 25 and 37 °C).

### Photometer linearity and precision

The linearity and precision of the photometer were estimated by replicate absorbance measurements of potassium dichromate solutions at 334 nm. The photometer was found to be linear at this wavelength between 0·01 and 2·5 absorbance units with coefficients of variation ranging from 2·0% at an absorbance of 0·01 U to 0·06% at an absorbance of 2·0 U.

### Overall system performance

The overall assessment of accuracy, precision, linearity and carry-over was made using three chemistries. These were: (1) urea using a UV kinetic urease/glutamate dehydrogenase method (Merkotest Urea [GLDH] MERCK 14343); (2) calcium by an o- cresophthalein complexone/2-amino-2-methylpropan-1-ol method using reagents supplied by BDH (BDH Chemicals Ltd, Broom Road, Poole BH12 4NN, Dorset, UK); (3) gamma-glutamyl transpeptidase (GGT) utilizing the liberation of 5-amino-2-nitrobenzoate from 2-γ-glutamyl-3-carboxy-4-nitroanilide (Merckotest GT, MERCK 14302). The recommended method parameters for each chemistry were programmed into the EPOS without modification from the method handbook except that the values of the standards were changed to suit the calibration material.

### *Precision*

Within-batch precisions were calculated each day for 20 days from the results of 12–14 replicate measurements on a serum pool prepared freshly each day. Between-day precisions were calculated for serum pools containing high, medium and low levels of analyte. Aliquots of each pool were stored frozen at −20 °C and a total of 23 replicate measurements were made over a 30-day period.

The results (tables 2[*a*] and [*b*]) show reasonably good analytical CVs for all three chemistries. Although these results were calculated after excluding outliers greater than 3 SDs from the mean, these occurred only twice amongst all the calcium results ($N = 236$), three times amongst the GGT results ($N = 278$) and never amongst the urea results ($N = 240$).

It was noticed that the precision of the calcium method decreased if the sample probe had already entered the sample cup one or more times. In an experiment to investigate this, samples were analysed repeatedly for calcium in 20 different sample cups. The within-batch imprecision doubled and was significantly different ($F = 3{\cdot}24$, $p < 0{\cdot}01$) after the probe had entered the sample cups five times (table 3). Although, initially, this appeared to be a problem caused by the sampling system,

*Table 2. Precision of analysis for calcium, urea, and GGT in serum pools.*

*(a) Within-batch precision (results are the average of 20 separate determinations of 12–14 replicate measurements).*

| Test | Mean | CV (%) |
|---|---|---|
| Calcium (mmol/l | 2·29 | 1·69 |
| Urea (mmol/l) | 6·06 | 3·09 |
| GGT (IU/l) | 56·02 | 1·22 |

*(b) Between-batch precision (23 replicate measurements made over a 30-day period).*

| Test | Mean | CV (%) |
|---|---|---|
| Calcium | 1·94 | 4·2 |
| (mmol/l) | 2·43 | 4·8 |
| | 3·29 | 2·8 |
| Urea | 6·06 | 6·8 |
| (mmol/l) | 14·01 | 6·2 |
| | 29·01 | 5·8 |
| GGT | 26 | 6·2 |
| (IU/l) | 114 | 6·2 |
| | 435 | 7·1 |

excessive sample to sample carry-over was not demonstrated and the other two chemistries did not show the same phenomenon. It is possible that the amount of time the samples were left in the cups was a contributory factor since it was also noticed that the precision deteriorated markedly after the samples had been left overnight in the cups (within-batch standard deviation [SD] = 0·027 mmol/l before storage; within-batch SD = 0·072 mmol/l after 18 h at 4 °C [$F = 7{\cdot}1$, $p < 0{\cdot}001$]). In order to avoid these problems, samples for calcium were analysed as soon as possible after transfer to the sample cups and if multiple analyses were required calcium was measured first.

### *Accuracy*

Accuracy of the instrument was assessed by two methods. Firstly, using patient samples, the methods for the three chemistries on the EPOS were compared with the methods in routine use in the laboratory. The comparative methods used were: (1) for urea an o-phthaldehyde/8-(4amino-1-methylbutamino)-6-methoxyquinoline method set up on an American Monitor Parallel Analyser (American Monitor [UK] Ltd, Robell Way, Storrington, West Sussex RH20 3DW, UK); (2) for calcium an o-cresophthalein complexone method using diethylamine buffer also set up on the Parallel; and (3) for GGT an in-house method utilizing the liberation and measurement of 5-amino-2-nitrobenzoate from L-glutamyl-3-carboxy-4-nitroanilide (2) set up on a Union Carbide Centrifichem. The patient samples were run in duplicate on both the EPOS and the Parallel, the mean value being used in the comparison. Regression analysis was by the Deming modification for two sets of variable data [3].

The results (figures 2, 3 and 4) showed very good agreements between methods for both urea and GGT but

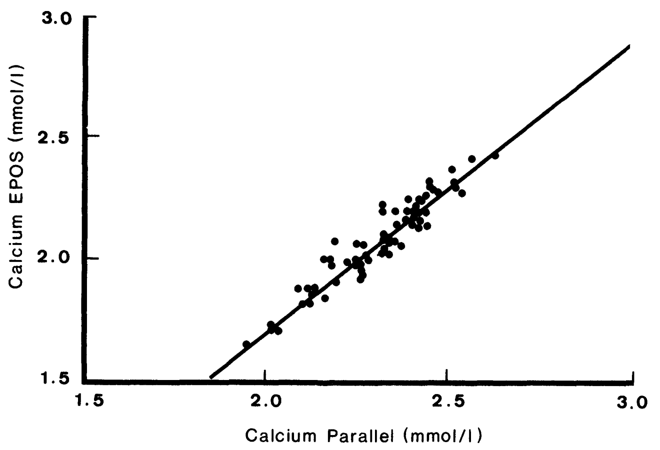

*Figure 2. Comparison of EPOS calcium results with results on the Parallel using patient samples. Orthogonal regression equation (Deming) y = 1·193x-0·693; correlation coefficient (r) = 0·948; N = 64.*

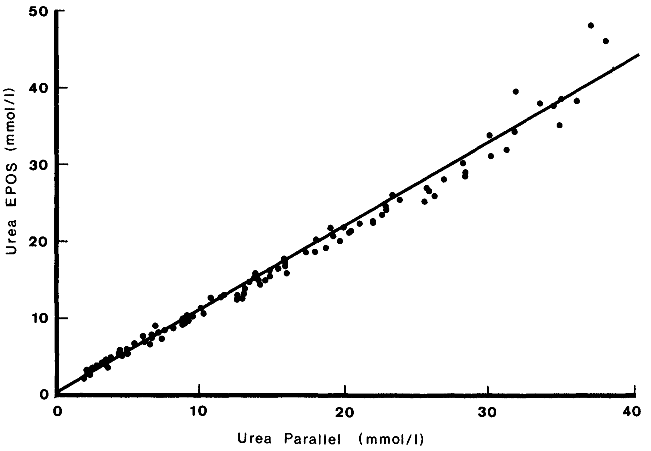

*Figure 3. Comparison of EPOS urea results with results on the Parallel using patient samples. Orthogonal regression equation (Deming) y = 1·086x-0·055; correlation coefficient (r) = 0·992; N = 97.*

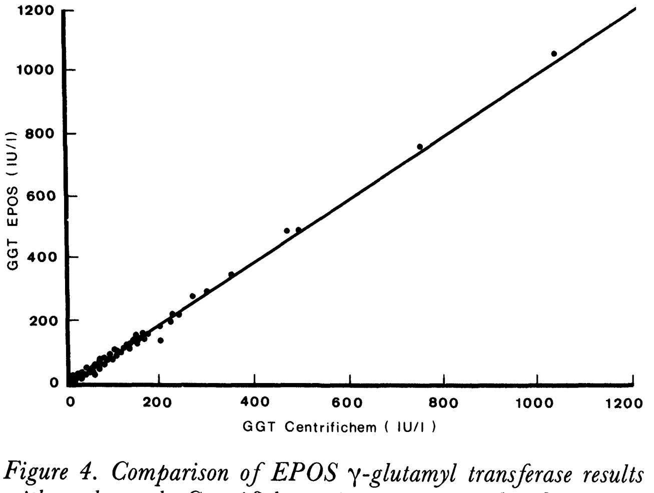

*Figure 4. Comparison of EPOS γ-glutamyl transferase results with results on the Centrifichem using patient samples. Orthogonal regression equation (Deming) y = 1·01x-0·088; correlation coefficient (r) = 0·999; N = 86.*

the comparison of the calcium methods showed a poor correlation with an unacceptable amount of scatter ($r$ = 0·948) and measured calcium concentrations between 0·2 and 0·3 mmol/l lower by the EPOS compared with the Parallel. The within-batch precisions for the two calcium methods calculated from the differences between duplicates were both good (EPOS; CV = 1·05%, Parallel; CV = 1·39%) which suggested that poor precision was not responsible. A more likely reason may have been the calibration of the method on the EPOS which did cause some problems. It was not possible to perform a two-point calibration at calcium concentrations of 1·5 and 3·0 mmol/l as the EPOS repeatedly rejected the calibration run with an error message indicating that the line from zero through the calibration points was non-linear. Consequently a single point calibration at a level of 2·82 mmol/l had to be used which may have been responsible for the bias seen in the comparison.

The second assessment of accuracy was performed by analysing a number of different control sera and comparing the results obtained for the three analytes with the manufacturer's or consensus values given for each one. The control sera used were: (*a*) Dade I and II (M+D QAP Chemistry Controls 150275 and 150276 supplied by American Hospital Supply [UK], Wallingford Road, Compton, Nr. Newbury, Berkshire RG16 QOW, UK); (*b*) Wellcomtrol Quality Control Sera BCB4 and BCB5 (Wellcome Reagents Ltd, Wellcome Research Laboratories, Beckenham BR3 3BS, Kent, UK); and (*c*) NEQAS reference serum Lot HIQC10 (UKEQAS, Wolfson Research Laboratories, Queen Elizabeth Medical Centre, Birmingham B15 2TH, UK). The results (table 4) are shown as the percentages of the assigned values and are means of at least 20 determinations. They show an overall positive bias for the urea and GGT methods whereas the calcium method has a positive bias at high levels and a negative bias at lower values. It seems likely that this was the result of using only a single point calibration at a level of 2·8 mmol/l as discussed previously.

*Table 3. Effect of repeated samplings on calcium within-batch precision. Variances were compared to that of the first sampling using the variance ratio (F) test. NS = not significant ($p < 0.05$).*

| Number of Samplings | Mean (mmol/l) | CV (%) | P |
|---|---|---|---|
| 1 | 2·31 | 0·64 | |
| 2 | 2·29 | 0·90 | NS |
| 3 | 2·30 | 1·40 | <0·001 |
| 4 | 2·29 | 0·88 | NS |
| 5 | 2·28 | 1·20 | <0·01 |
| | 18 h at 4°C | | |
| 6 | 2·26 | 3·20 | <0·001 |

*Linearity*

The linearity of the calcium method was determined by measuring the end-point absorbances of the reaction mixture in a series of dilutions made from a specimen with a high calcium concentration (3·3 mmol/l). The line passing through these points was compared with the calibration line drawn between the origin and the absorbance value of the calibration standard (figure 5).

*Table 4. Comparison of consensus/manufacturers' values and measured values for five different control sera. Control sera were: A = M+D Dade 1; B = M+D Dade 2; C = Wellcomtrol BCB5; D = Wellcomtrol BCB4; E = NEQAS HIQC 10.*

| TEST | | Reference Sera | | | | |
|---|---|---|---|---|---|---|
| | | A | B | C | D | E |
| Calcium | Consensus value | 2·31 | 3·24 | 2·24 | 2·82 | 2·82 |
| | Measured value | 2·28 | 3·29 | 2·21 | 2·87 | 2·93 |
| | % found | 98·7 | 101·5 | 98·7 | 102·5 | 103·9 |
| Urea | Consensus value | 5·2 | 17·9 | 6·1 | 17·0 | 7·0 |
| | Measured value | 5·7 | 18·6 | 6·3 | 17·8 | 7·4 |
| | % found | 109·6 | 103·9 | 103·3 | 104·7 | 105·7 |
| GGT | Consensus value | 34 | 112 | - | - | - |
| | Measured value | 36 | 108 | - | - | - |
| | % found | 105·9 | 103·6 | - | - | - |

This showed the method to be linear over the range 1·5 mmol/l to 3·3 mmol/l with a positive bias at levels below 1·5 mmol/l.

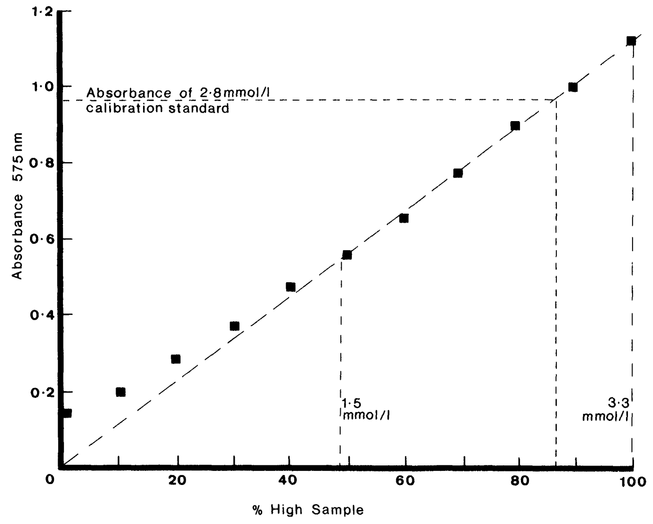

*Figure 5. Linearity of the EPOS calcium method. Absorbances of dilutions of a high value specimen (■) compared with the calibration line derived from a one-point calibration at 2·8 mmol/l (----).*

As it was not possible to measure directly the changes in absorbance for the urea and GGT methods the linearity was assessed by measuring the levels of analyte in a series of dilutions of high value specimens. The GGT method was linear up to 1000 IU/l and the urea method up to 45 mmol/l (figures 6 and 7).

*Carry-over*

The effect of sample to sample carry-over by the sample probe was assessed by measuring successive samples of serum with a high concentration of analyte followed by three samples of serum with a low concentration using the proceedure described by Broughton *et al.* [1]. The mean carry-overs from 10 separate determinations were 0·57, 1·7 and 1·2% for the GGT, urea and calcium methods respectively.

### Running costs

The most time-consuming operation when performing an analysis on the EPOS is the aliquoting of specimens into the sample cups, but once a batch has been set up a single operator can run a wide range of tests on a relatively large number of samples (up to 200 per batch). This is helped by the ability to run different chemistries on selected samples without having to remove them from the sample chain and a facility which automatically repeats, on dilution, specimens with high levels of analyte. Changing between chemistries simply requires the preparation of new reagents. This can be carried out beforehand in new

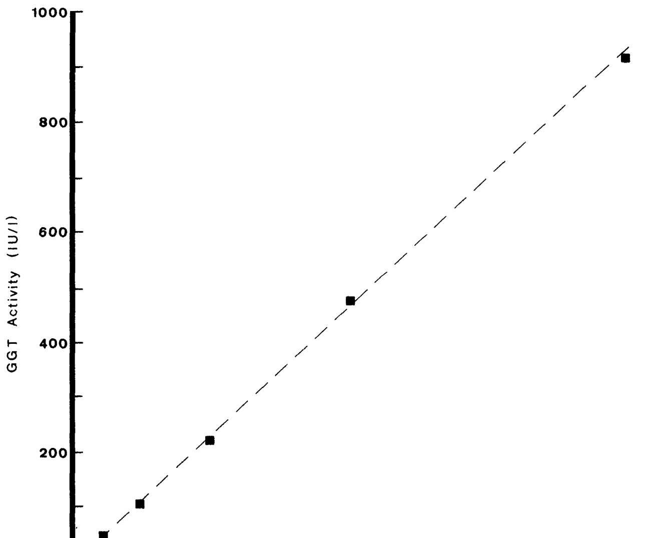

*Figure 6. Linearity of the EPOS γ-glutamyl transferase method.*

reagent reservoirs so that they are ready to exchange with those from the previous analysis as soon as it is over. Once a method has been set up, no operator intervention should be necessary until the batch is completed.

Because the reaction cuvettes are an integral part of the instrument, and are laundered during the rotor cycle, consumables for the operation of the EPOS are kept to a minimum. The main costs incurred are in the disposable sample cups, the 'system solution' which is used as a wetting agent in the rinsing and flushing water, and the detergent solution which is used to wash the cuvettes at the end of each batch of specimens. Parts such as new probes and tubing should not need to be replaced very frequently and are therefore not included in the running costs here.

With an instrument of this type reagent costs will vary depending upon how it is used, i.e. whether it is operated as a high throughput batch analyser or as a specialist analyser for small batches of less frequently used tests. In addition, much will depend upon whether commercial kits or 'in-house' methods with individually bought reagents are used. However, the reagent dispensing system of the EPOS ensures that whichever is used reagent wastage is minimized since it is possible to make up only the required amount of reagent for the number of analyses to be done. Also there is a very low dead volume requirement: the R1 reservoir has a minimum dead volume of 1 ml and the R2 reservoir one of 250 µl.

An analysis of the overall cost of consumables and reagents per test for the three chemistries that were evaluated gave figures of £0·021 for calcium, £0·069 for urea and £0·062 for GGT.

### Reliability and safety

During the two months in which the EPOS was in use in the laboratory there were no major malfunctions during operation. On two occasions the polypropylene R1 probe was found to have bent during operation, most likely by it hitting the side of the washing well into which it dips after dispensing reagent into the cuvettes. On each occasion the batch was aborted and restarted after replacement of the probe. On another occasion the metal sample probe was irreversibly buckled but this was due to the use of the incorrect type of sample cup with a too thick cap.

An intermittent problem which occurred three times during the evaluation period involved the instrument spontaneously aborting a run and displaying an error message as soon as it had sampled from the first sample cup. In each case the error message indicated a horizontal displacement of the sample probe so that it was mis-aligned with respect to entry into the cuvette. However, inspection showed no perceptible displacement of the probe when it was returned to its resting position. Usually the problem was resolved by resetting the instrument although on one occasion the same problem recurred four times before spontaneously resolving.

One of the features of the EPOS is that it requires very little maintenance. In practice it was found that the only

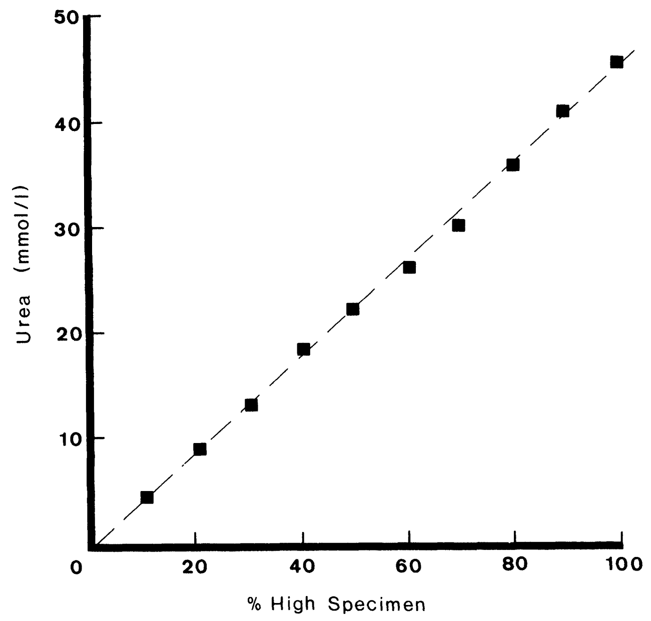

*Figure 7. Linearity of the EPOS urea method.*

regular daily maintenance necessary was the priming of the syringes to expel air bubbles which were a frequent problem even though the rinsing and flushing solution contains a wetting agent. The use of glass distilled rather than deionized water as in this case should alleviate this problem. Regular weekly maintenance was limited to soaking the cuvettes in detergent solution overnight to thoroughly clean them and wiping of the probes with organic solvent to remove accumulated serum constituents. All other maintenance such as emptying of the waste container, filling the rinse solution reservoir and replacing bent or broken probes was carried out as required.

With respect to overall mechanical and electrical safety the instrument was found to be well designed. The majority of the electrical and mechanical systems were well contained and only accessible by unscrewing one or more of the covering panels. The external moving parts were shielded during operation which prevented the chance of accidental injury by the sample or reagent probes. Although the rotor was accessible during operation its flush fit into the body of the instrument meant that there was little danger of it catching anything whilst it was moving. There were no obvious biological hazards evident during operation, mainly due to the fact that the sample is aspirated from a covered sample cup and deposited well down inside the cuvette. All the waste from the cuvettes is treated with ultra-violet light before draining to the waste container which has a capacity of 5 l, enough for the waste from 700 analyses. When full it must be emptied and, because of its shape and the position of the drain hole, care must be taken to avoid excessive spillage when pouring the liquid away.

### Subjective assessment

After two months' experience with the EPOS, the overall opinion was that it was a well designed and manufactured instrument. It took little more than a day to master the fundamentals of its operation and after this the performance of any programmed chemistry was simple. This was helped by a number of useful features such as the automatic repeat on dilution of high value specimens, the ability to designate any number of samples in a batch to be automatically repeated, and the ability to set up a worklist using the keyboard to assign samples to be analysed for one or more additional chemistries.

A very useful feature was the menu of manual functions which provided a facility for method investigation and development and in this case was helpful for performing the mechanical evaluation. The handbook provided with the instrument competently described the use of all the keyboard functions. In addition, a methods handbook was provided which contained details of all the parameters necessary to set up methods based on the use of commercial kit reagents. However, it was found to be relatively easy to adapt in-house methods to run on the EPOS.

Because there is no positive sample identification system within the instrument's software, problems of sample identification after they have been dispensed into the sample cups could arise if the instrument were used for high sample throughput analysis without an external data-handling facility. This facility is available as an optional extra to prepare worklists and assign tests to samples.

Initial doubts about the performance of the photometer due to its openness to stray light and the problems of dust and other matter contaminating the uncovered cuvettes were unfounded with experience. Indeed, in a recent study in which light was shone directly into the measuring cuvette no significant effect on the photometer was noticed [4].

Apart from one universal complaint in the laboratory concerning the annoying noise that the rotor made during operation — a jarring, grating noise — the overall impression was that the EPOS was aesthetically pleasing to work with. Because of its versatility the EPOS should find a role in many laboratories, whether as the major analytical instrument in a smaller laboratory or as a back up instrument with specialist method facilities in others.

### Acknowledgements

The authors would like to thank B. D. H. Diagnostics for the load of the EPOS for the period of the evaluation and for their advice and support. They would also like to thank Mr R. W. Richardson for the use of laboratory facilities and Eppendorf for permission to reprint figure 1.

### References

1. Broughton, P. M. G., Gowenlock, A. H., Mccormack, J. J., and Neill, D. W., *Annals of Clinical Biochemistry*, **11** (1974), 207.
2. IFCC Methods for the Measurement of Catalytic Concentrations of Enzymes Part 4. IFCC Method for Glutamyltransferase, *Journal of Clinical Chemistry and Clinical Biochemistry*, **21** (1983), 633.
3. Deming, W. E., in *Statistical Adjustment of Data* (John Wiley, New York, 1943), p. 184.
4. Moses, G. C., Lightle, G. O., Tuckerman, J. F. and Henderson, A. R., *Clinical Chemistry*, **32** (1986), 165

### NOTES FOR AUTHORS

*Journal of Automatic Chemistry incorporating Journal of Clinical Laboratory Automation* covers all aspects of automation and mechanization in analytical, clinical and industrial environments. The *Journal* publishes original research papers; short communications on innovations, techniques and instrumentation, or current research in progress; reports on recent commercial developments; and meeting reports, book reviews and information on forthcoming events. All research papers are refereed.

### Manuscripts

Two copies of articles should be submitted. All articles should be typed in double spacing with ample margins, on one side of the paper only. The following items should be sent: (1) a title-page including a brief and informative title, avoiding the word 'new' and its synonyms; a full list of authors with their affiliations and full addresses; (2) an abstract of about 250 words; (3) the main text; (4) appendices (if any); (5) references; (6) tables, each table on a separate sheet and accompanied by a caption; (7) illustrations (diagrams, drawings and photographs) numbered in a single sequence from 1 upwards and with the author's name on the back of every illustration; captions to illustrations should be typed on a separate sheet. Papers are accepted for publication on condition that they have been submitted only to this *Journal*.

### References

References should be indicated in the text by numbers following the author's name, i.e. Skeggs [6]. In the reference section they should be arranged thus:

to a journal

Manks, D. P., *Journal of Automatic Chemistry*, **3** (1981), 119.

to a book

Malmstadt, H. V., in *Topics in Automatic Chemistry*, Ed. Stockwell, P. B. and Foreman, J. K. (Horwood, Chichester, 1978), p. 68.

### Illustrations

Original copies of diagrams and drawings should be supplied, and should be drawn to be suitable for reduction to the page or column width of the *Journal*, i.e. to 85 mm or 179 mm, with special attention to lettering size. Photographs may be sent as glossy prints or as negatives.

### Proofs and offprints

The principal or corresponding author will be sent proofs for checking and will receive 50 offprints free of charge. Additional offprints may be ordered on a form which accompanies the proofs.

*Manuscripts should be sent to either Dr P. B. Stockwell or Ms M. R. Stewart, see inside front cover.*

